# Label-Free Measurement of Ligand Interactions Using SABRE Hyperpolarization at Low Magnetic Fields

**DOI:** 10.1021/acs.analchem.5c07983

**Published:** 2026-03-10

**Authors:** Ashes Roy, Christian Hilty

**Affiliations:** Chemistry Department, Texas A&M University, College Station, Texas 77843, United States

## Abstract

The protein–ligand binding affinity is evaluated using low-cost, low-field nuclear magnetic resonance (NMR) spectroscopy at 0.85 mT. Strong signals are achieved through the hyperpolarization of ^1^H nuclei by parahydrogen-based signal amplification by reversible exchange (SABRE). The interaction is monitored by tracking the ubiquitous hydrogen signal. Despite the hyperpolarization, a key challenge of the label-free detection at low field is the distinction of signals in the absence of chemical shift. ^1^H signals of ligands are measured after deuterating the coligand and solvent and numerically accounting for orthohydrogen signal, which is produced during the hyperpolarization process. Spin–spin (*R*_2_) relaxation rates quantify the protein–ligand interaction. Since *R*_2_ in a milli-Tesla field does not include an exchange contribution, the calculation of the ligand dissociation constant *K*_D_ is simplified compared to traditional high-field NMR. The technique for monitoring ^1^H signals is generalizable to detect any ligand that is competitively binding to the protein. It expands the application of low-field NMR for high-throughput screening and for studies of biochemical processes that involve ligand interactions.

## Graphical abstract

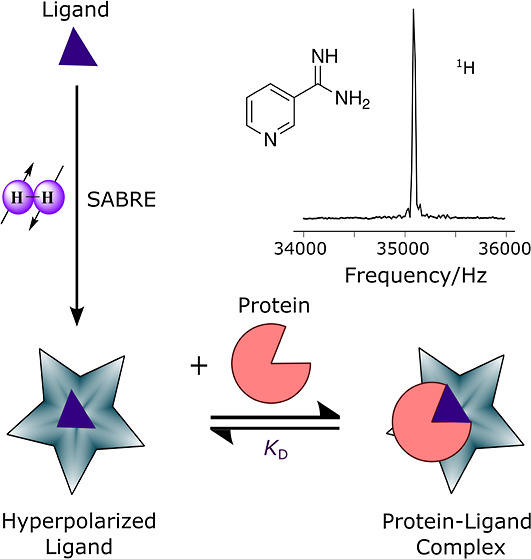

## 1 Introduction

Protein and ligand interactions are essential for transduction of signals, gene regulation, and numerous other biological functions.^1,2^ Their measurement is required for drug discovery and in basic investigations of mechanisms of biological regulation and related processes. Nuclear magnetic resonance (NMR) spectroscopy is uniquely positioned to characterize these interactions by enabling the observation of ligand-specific signals that experience a change upon binding in a solution environment. The observable parameters include chemical shifts, relaxation rates of various nuclear spin states, self-diffusion coefficients, and others.^3,4^

Traditionally, biomolecular NMR is performed at a high magnetic field of up to 20 T or more, where high-resolution spectra suitable for the determination of macromolecular structures can be measured. The questions related to ligand interactions are less complex, in some cases only asking whether a ligand binds to the protein. In other cases, measurement of binding affinity is required. Despite the removal of the need for high resolution for these measurements, ligand interactions are still most commonly studied using the same high-field NMR instruments that are optimized for structural studies. This trend is true even in an industrial setting, where small-molecule compounds are routinely screened for drug discovery.^5,6^

Recent developments in nuclear spin hyperpolarization enable the measurement of strong NMR signals from biological small molecules without requiring a high magnetic field. The spin polarization that is normally achieved by equilibration of the spin system in a high magnetic field is replaced by a large hyperpolarization produced prior to the NMR experiment. A low-field magnet can be used to perform NMR spectroscopy of biomolecular samples for cost efficiency and experimental simplicity. Low-field NMR presents an additional significant advantage in simplifying the interpretation of *R*_2_ spin relaxation rates because there is no exchange contribution to the relaxation in the absence of chemical shift.^7^

The hyperpolarization of biological molecules for liquid-state NMR has been demonstrated by several techniques, foremost dissolution dynamic nuclear polarization (D-DNP),^8^ chemically induced dynamic nuclear polarization (CIDNP),^9^ and parahydrogen-induced polarization (PHIP).^10,11^ D-DNP is capable of hyperpolarizing various nuclear spins, such as ^13^C, ^1^H, ^19^F, or ^31^P through a microwave-based pumping mechanism starting from electron spin polarization. Most biological small molecules are compatible with D-DNP, which holds promise as a universal hyperpolarization technique. Since the DNP polarizer requires a superconducting magnet itself, it is most naturally applied in combination with high-field NMR.

Other hyperpolarization methods are not subject to this requirement. CIDNP provides a sensitive means for studying ligands that can undergo a photochemical reaction with a photosensitizer.^12^ Because CIDNP requires only the addition of a light source to an NMR spectrometer, it can generate polarization at low cost. A similar advantage is realized by PHIP. Parahydrogen, the antiparallel nuclear spin state of molecular hydrogen, can be produced by cryogenically cooling hydrogen gas. The para spin state is stable for extended time periods. It can be converted into hyperpolarization through hydrogenative or nonhydrogenative chemical processes. The signal amplification by reversible exchange (SABRE) technique hyperpolarizes a ligand that binds with an iridium center to form a polarization transfer complex, without changing the chemical identity of the ligand. SABRE requires a moiety such as pyridine that contains electron density to bind to the metal,^13–17^ while hydride signals in similar complexes can be used to indirectly sense the presence of biological molecules.^18^ When mixed with a protein solution in a high-field NMR spectrometer, the binding of a SABRE hyperpolarized ligand can be identified by a change in *R*_2_ relaxation.^19,20^ Since the SABRE polarization occurs in the milli-Tesla range, it is ideally compatible with NMR spectroscopy at the same magnetic field. Low-field NMR with SABRE in homogeneous electromagnets is amenable to detect signals of different types of nuclei, such as ^1^H, ^13^C, ^15^N, and ^19^F, in a single scan.^21–24^ Our group has previously shown that the interaction between a protein and a ligand can be quantified by monitoring a fluorine signal.^7,25–27^

Here, we measure the binding of label-free ligands using SABRE hyperpolarization of ^1^H, abundant in biological molecules. We show that low-field NMR is advantageous for a simplified calculation of the binding affinity because an exchange contribution to the *R*_2_ relaxation is absent. On this basis, we demonstrate the measurement of binding affinities with a SABRE hyperpolarized reporter ligand.

## 2 Experimental Section

### 2.1 Sample Preparation

The samples for all the experiments performed in the low- and high-field magnets contained chloro­(1,5-cyclooctadiene)­[4,5-dimethyl-1,3-bis­(2,4,6-trimethylphenyl)­imidazole-2-ylidene]­iridium­(I) as the precatalyst (C) (Strem, Newburyport, MA), 3-amidino-pyridine hydrochloride as the reporter ligand (L) (Advanced Chemical Intermediates, Davidstow, Cornwall, U.K.), and deuterated dimethyl sulfoxide (DMSO-*d*_6_) as the coligand (CoL) (Cambridge Isotope Laboratories, Andover, MA) in deuterated methanol (*d*_4_-methanol) (Cambridge Isotope Laboratories, Andover, MA) at the concentration ratio of 1:10:10. Solutions of trypsin protein were prepared freshly for the experiments by dissolving 40 ± 2 μM trypsin from bovine pancreas (Alfa Aesar, Ward Hill, MA) in 50 mM phosphate buffer (NaH_2_PO_4_ and NaH_2_PO_4_) at pH 7.2. The trypsin concentration was determined by UV spectrophotometry (ε_280_ = 35.1 cm^–1^ mM^–1^).^19^ 17.28 ± 0.864 mM of benzamidine or benzylamine was added to the protein solution for the competing experiments. For the catalyst–ligand binding experiments, the concentrations of L and CoL were kept constant at 10.00 ± 0.50 mM, and the catalyst concentration was 1.00 ± 0.05, 5.00 ± 0.25, or 10.00 ± 0.50 mM. For the protein binding experiments, 1.25 mL of trypsin solution was mixed with 0.25 mL of hyperpolarized sample, diluting the hyperpolarized sample 6 times and changing the concentration of the injected solution to 5/6th of the initial value. For protein binding experiments, the initial concentrations of the hyperpolarized sample were 1.00 ± 0.05 mM C, 10.00 ± 0.50 mM L, and 10.00 ± 0.50 mM CoL, whereas the final concentrations were 0.167 ± 0.083 mM C, 1.67 ± 0.83 mM L, and 1.67 ± 0.83 mM CoL. The final concentrations of the trypsin protein and benzamidine or benzylamine competitor ligands were 33.33 ± 1.67 μM and 14.40 ± 0.72 mM, respectively.

### 2.2 NMR Spectroscopy

The low-field NMR spectrometer was configured as previously described.^25^ Briefly, an electromagnet provided a magnetic field of 0.85 mT, resulting in a ^1^H Larmor frequency of 35,240 Hz. Figure S1a represents the experimental setup. The NMR tube is placed inside of an RF coil, a SABRE field coil, and a pulse coil enclosed in an aluminum shield. The magnetic field is produced by a tetracoil located outside of the shield. A syringe pump is used to inject and mix the protein sample with the hyperpolarized sample. The mixing was verified visually in test experiments using colored solutions. The relaxation experiments were divided into two groups. The first one comprised catalyst–ligand binding experiments. In the second, protein was rapidly injected into the hyperpolarized sample by using the syringe pump to measure protein binding. The parahydrogen was enriched using a closed-cycle cryocooler (Advanced Research Systems, Macungie, PA) at 29 K. The catalyst was activated with parahydrogen at 298 K for 5 min. The sample was pressurized at 120 psi in a 10 mm NMR tube. During the experiment, parahydrogen was bubbled into the sample for a time *t*_1_ of 20–30 s with a flow rate of 0.2 slpm (Figure S1b). The magnetic field was stepped up to 5 mT during this time by using the SABRE field coil. The bubbling was stopped 0.2 s prior to turning off the SABRE field. The protein was injected with a flow rate of 114 mL/min. A prescan delay of *t*_3_ = 0.4 s allowed for mixing. Subsequently, a pulse sequence consisting of p90–τ [2·p90–2·τ]_×*n*_ was applied for the *R*_2_ relaxation measurement. The delay τ was 50 ms, the pulse length p90 = 0.35 ms, and the echo number *n* = 100. The phase of the 90° pulse was *x* and of the 180° pulses was *y* (Figure S1b). For the calculation of [CL]/[L] ratio, ^1^H NMR spectra were measured at different catalyst concentrations in a 400 MHz NMR spectrometer (Bruker Biospin, Billerica, MA) using a 5 mm triple resonance (TXI) probe.

### 2.3 Data Analysis

Each spin echo was Fourier-transformed, and the peak from each was integrated (Figure S2). An exponential curve was fitted to the integrated values to determine the *R*_2,obs_. The relaxation data in each case were fitted with the corresponding model for the equilibrium concentrations by minimizing the residual sum of squares in Python. Bounds were applied for concentrations to not exceed their initial values in the fits for dissociation constant measurements. A Monte Carlo analysis was used to determine the mean and confidence ranges of the fitted parameters. Since the resulting distributions generally were nonsymmetric, the lower and upper bounds of the confidence ranges were calculated as the narrowest regions containing 90% of the values.

## 3 Results and Discussion

Proton NMR spectra of a SABRE hyperpolarized ligand, 3-amidino-pyridine, are shown in Figure 1. Strong signals are observed both in a conventional high-field magnet at 9.4 T and in an electromagnet operated at 0.82 mT. In both cases, spin polarization is governed by the SABRE process, independent of the measurement field. In the high field, signals from the four aromatic protons are observed (Figure 1a). A comparison with a nonhyperpolarized spectrum reveals signal enhancements of up to 461-fold. Additionally, a signal from orthohydrogen, which is produced by the SABRE process, is visible. Solvent signals would also appear, although these are mostly suppressed due to the use of deuteration in this sample. In contrast, at 0.82 mT, only a single ^1^H signal is observed (Figure 1b). In this magnetic field regime, chemical shift differences are much smaller than the line width and are not resolved. Additionally, homonuclear coupling constants are in the strong coupling regime and therefore result in only a single peak.

**Figure 1:**
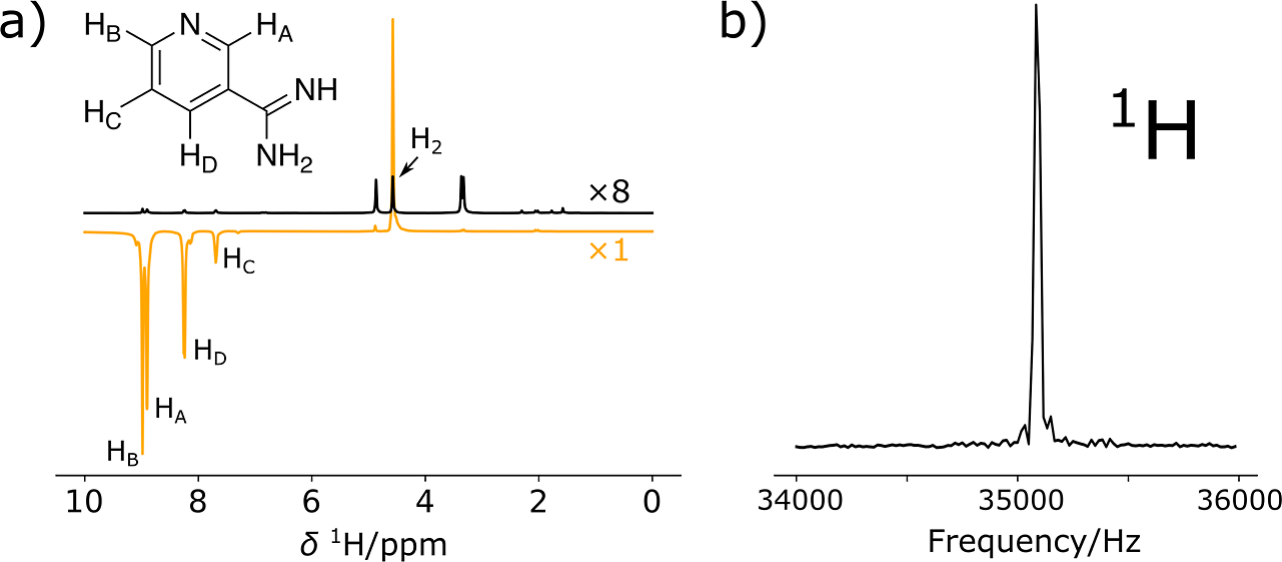
(a) ^1^H hyperpolarized NMR spectrum (orange line) vs 8× magnified nonhyperpolarized spectrum (black line) of 3-amidinopyridine measured at a magnetic field of 9.4 T (400 MHz Larmor frequency). (b) Hyperpolarized spectrum of the same sample measured at 0.82 mT (35.2 kHz frequency), showing signal enhancements of 461, 398, 381, and 91 for the *H*_B_, *H*_A_, *H*_D,_ and *H*_C_, respectively. In both cases, the sample contained 1.000 ± 0.050 mM of chloro­(1,5-cyclooctadiene)­[4,5-dimethyl-1,3-bis­(2,4,6-trimethylphenyl)­imidazole-2-ylidene]­iridium­(I) as the precatalyst (C), 10.00 ± 0.50 mM of 3-amidinopyridine hydrochloride as the ligand (L), and 10.00 ± 0.50 mM of DMSO-*d*_6_ as the coligand (CoL) in *d*_4_-methanol. Sample volumes were 0.5 mL in (a) and 0.25 mL in (b).

In the following, this single ^1^H NMR signal is intended to be used for determining the interaction of the ligand with a target protein mixed with the hyperpolarized sample. The interaction is quantified by the *R*_2_ relaxation measured from the exponential decay of a series of spin echoes, each of which is individually Fourier-transformed (Figure 2). In the absence of labeling the molecule of interest with another spin that would provide a unique signal, the detection of the molecule poses a challenge due to the inability to distinguish the ^1^H signals of the various chemical species present. As a result, a clearly nonexponential time dependence is observed, which does not allow the determination of *R*_2_ (Figure 2a). This signal course is expected to be due to multiple simultaneous spin polarization processes. First, SABRE polarizes protons of the ligand, the coligand, dimethyl sulfoxide, and possibly the methanol solvent, all of which are protonated. Cross-relaxation then redistributes the polarization among the species present.^28^ Additionally, the SABRE process converts parahydrogen to observable orthohydrogen. In Figure 2b, the nonexponential behavior is alleviated by deuteration of the solvent and coligand to prevent contribution of other molecules apart from the ligand to the proton signal. The resulting smooth exponential decay curve allows for the determination of an *R*_2_ parameter characterizing the decay. Orthohydrogen still contributes to this signal, as described at the end of the text.

**Figure 2:**
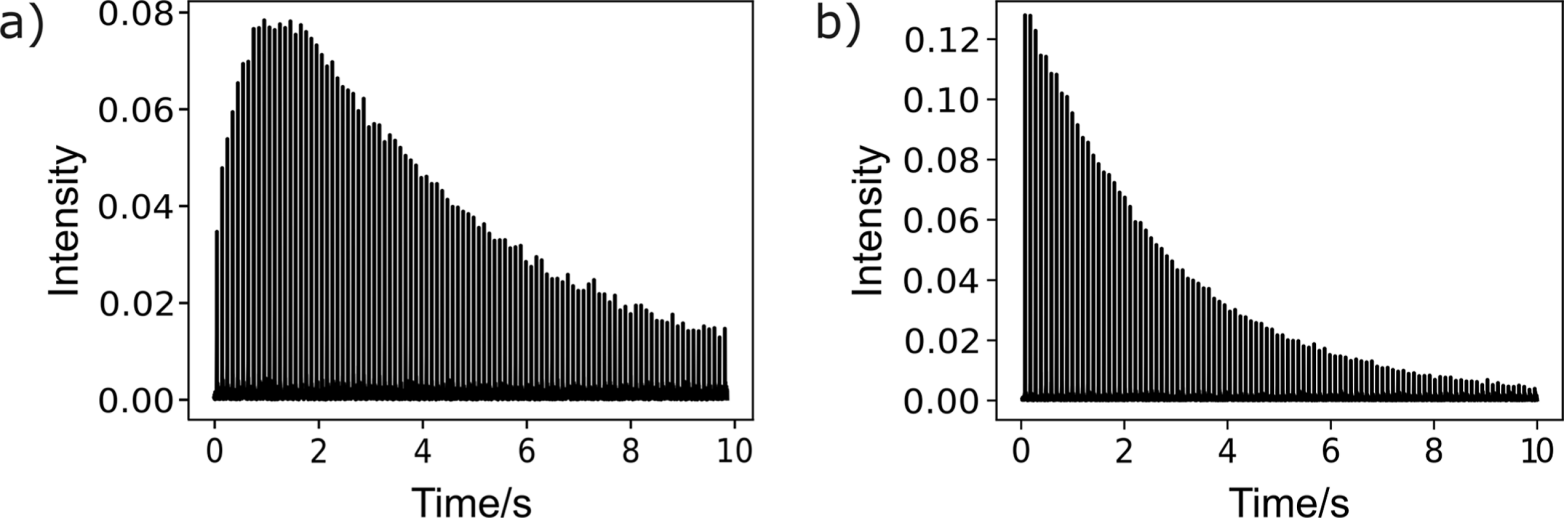
(a) Frequency domain signals from 1.000 ± 0.050 mM C, 10.00 ± 0.50 mM L, and 10.00 ± 0.50 mM of DMSO as the coligand in methanol. (b) Frequency domain signals for the sample containing C, L and 10.00 ± 0.50 mM of DMSO-*d*_6_ as the coligand (CoL) in *d*_4_-methanol.

The *R*_2_ rates are used in the following discussion to determine the protein–ligand interaction. First, the effect of catalyst and protein binding on the *R*_2_ relaxation rate of the ligand is determined. Subsequently, the hyperpolarized ligand is used as a reporter to determine the *K*_D_ of other ligands.

### 3.1 Ligand–Catalyst Binding

The binding of the ligand to the polarization transfer catalyst is described by

1 $$C+L\overset{K_{eq}}{⇄}CL$$

This equilibrium changes the concentration of the free ligand that will ultimately be available to interact with the protein. The relaxation rates of the free and catalyst-bound ligands, *R*_2,f_ and *R*_2,CL_, were determined by titrating the catalyst concentration (Figure 3 and S3). Notably, the observed *R*_2_ rates in the range of 0.3–1 s^–1^ for the ligand interacting with the catalyst are lower than those expected at high field. For example, an *R*_2_ of 2.40 s^–1^ was previously observed at 9.4 T for a similar ligand, 4-amidinopyridine, in the presence of 1.27 mM of an iridium complex.^19^ The *R*_2_ at the low field is not affected by an otherwise potentially dominant exchange line broadening.^7^ Instead, it reflects the properties of the bound form with a longer rotational correlation time (τ_c_).

**Figure 3:**
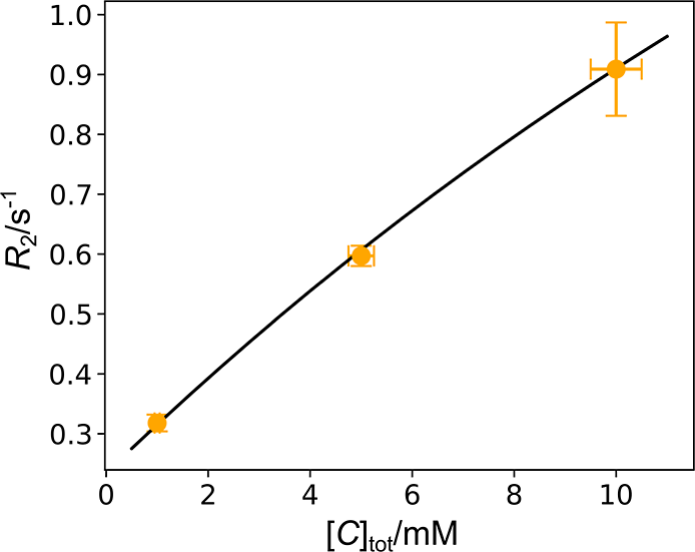
*R*_2_ relaxation rates of catalyst–ligand mixtures. [L_tot_] and [CoL] were fixed at 10.00 ± 0.50 mM. The data points are the measured *R*_2,obs_ (Figure S3). The numerical fitting described in the text resulted in *R*_2,f_ = 0.231 (0.17–0.28) s^–1^, *R*_2,CL_ = 2.92 (1.93–3.85) s^–1^, and *K*_eq_ = 0.050 (0.028–0.079) mM^–1^. The values are shown as average and 90% confidence interval from the Monte Carlo analysis. The black curve is back-calculated from these values.

The observed relaxation rates were fitted numerically by

2 $$R_{2,obs}=\frac{\left[CL\right]}{\left[L_{tot}\right]}R_{2,CL}+\frac{\left[L\right]}{\left[L_{tot}\right]}R_{2,f}$$

along with eqs S1–S3. These equations assume that only one ligand binds to the catalyst, whereby other coordination sites are occupied by the deuterated dimethyl sulfoxide used as a coligand.^29,30^ The calculation additionally requires knowledge of the ratio of the catalyst-bound ligand to the ligand concentration ([CL]/[L]), which was determined using high-field NMR (Table S1 and Figure S4).

### 3.2 Ligand–Protein Binding

Injecting protein into the solution of catalyst and ligand after the hyperpolarization process results in an additional relaxation change due to the protein binding equilibrium

3 $$P+L\underset{K_{D}}{⇄}PL$$

with the dissociation constant *K*_D_. If the ligand solution is mixed with phosphate buffer in the absence of protein, comparing the rates in Figure S3a–c and the first bar of Figure 4 indicates only small differences in the observed relaxation. A significant increase in *R*_2,obs_ in the presence of the protein trypsin (second bar in Figure 4) is indicative of the binding interaction. The increased *R*_2,obs_ is due to the relaxation rate of the protein-bound ligand, *R*_2,PL_, which can dominate eq 4 even when the fraction [PL]/[L_tot_] is only several percent.

4 $$R_{2,obs}=\frac{\left[PL\right]}{\left[L_{tot}\right]}R_{2,PL}+\frac{\left[CL\right]}{\left[L_{tot}\right]}R_{2,CL}+\frac{\left[L\right]}{\left[L_{tot}\right]}R_{2,f}$$

**Figure 4:**
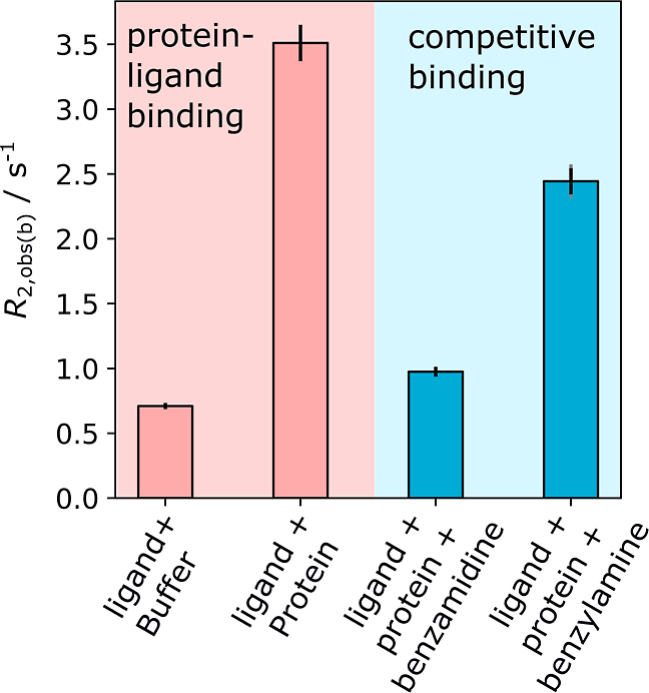
Observed *R*_2_ relaxation rates of protein binding experiments where the protein trypsin and competitor ligands were mixed with a sample containing 1.000 ± 0.050 mM C, 10.00 ± 0.50 mM L, and 10.00 ± 0.50 mM CoL to obtain the final concentrations 0.1667 ± 0.0080 mM C, 1.667 ± 0.080 mM L, and 1.667 ± 0.080 mM CoL. Relaxation data are shown in Figures S5 and S6. Error bars are standard deviations from three measurements (black) and propagated fitting errors (gray); shown only if larger.

The relaxation change upon addition of the protein is much larger than the contribution of the catalyst interaction, *R*_2,CL_, making it possible to directly observe the protein binding. In contrast, high-field NMR required the use of a capping ligand to inactivate the catalyst before the interaction with the protein was revealed.^19^ The relaxation change proves that the ligand binds to the protein but does not by itself provide a quantitative binding affinity.

### 3.3 Competitive Ligand–Protein Binding

The measurement of ligand binding affinity can be achieved in a competition experiment with a reporter ligand (L).^27^ A reporter ligand that is hyperpolarizable by parahydrogen is displaced from the binding site of the protein by a competing ligand of interest (S)

5 $$P+S\underset{K_{D,2}}{⇄}PS$$

The equilibrium in eq 5 is in addition to that in eq 3. With knowledge of the *K*_D_ of the reporter ligand, the *K*_D,2_ value of the competitor can be determined, or vice versa.^20,25^ Here, the ligand 3-amidinopyridine is intended to be used as a reporter, since its nitrogen-containing aromatic ring makes it readily hyperpolarizable by SABRE. Two competing ligands are benzamidine and benzylamine. In both cases, a slower relaxation rate of the reporter is observed in their presence (third and fourth bars in Figure 4). From the relaxation difference, it can be seen that benzamidine is a stronger competing ligand than benzylamine.

Because the *K*_D_ of the reporter ligand was not previously known, it was first determined using the affinity of benzamidine known from conventional NMR (50 mM Tris, 100 mM NaCl and 5 mM CaCl_2_, pH 8.0).^27^ The observed relaxation rates of 3-amidinopyridine (L) with trypsin and L with trypsin and benzamidine (S) shown in the second and third bars of Figure 4 were fitted. The eqs 3–5 together with the binding equilibria (eqs S2–S8) and the *R*_2,f_, *R*_2,CL_, and *K*_eq_ (Figure 3) were used. As a result, the dissociation constant of the reporter ligand and trypsin was determined as *K*_D_ = 141.1 (138.9–143.1) μM. This value is close to the *K*_D_ of a similar ligand, 5-fluoropyridine-3-carboximidamide (FCPA), which was reported in the range of 179 ± 12 μM.^25^

Finally, we demonstrate the measurement of the dissociation constant *K*_D,2_ of benzylamine using a competition with the reporter ligand. The relaxation measurement of the reporter ligand (L) in competition with benzylamine (fourth bar in Figure 4) was used along with the above-determined *K*_D_. The dissociation constant of benzylamine was thus determined as *K*_D,2_ = 170 (140–154) μM (eqs 4 and 5, S2–S8). The mean value outside the confidence region is due to a small number of divergent output values in the Monte Carlo calculation. The calculation of the *K*_D,2_ value demonstrates the utility of the low-field relaxation measurement of a reporter ligand to screen the interaction of other ligands with a protein.

The *K*_D_ of the benzylamine ligand is 238 ± 36 μM, determined using DNP hyperpolarization and conventional NMR (50 mM Tris, 100 mM NaCl and 5 mM CaCl_2_, pH 8.0)^27,31^ and 200 ± 80 μM using a high-field NMR competition experiment with a parahydrogen hyperpolarized reporter (45 mM phosphate, 10% MeOH, pH 7.6).^20^ Here (42 mM phosphate, 16% MeOH, pH 7.2), a somewhat lower value of 170 (140–154) μM was measured. The higher affinity could be speculated to continue a trend with increasing methanol concentration in the solution, although the differences are close to the uncertainty ranges.

### 3.4 Signal from Orthohydrogen

While the deuteration of the sample components eliminated the signals from these compounds, protons of the H_2_ gas are potentially observable. The hyperpolarization of the sample observed in the high-field NMR instrument shows that the ligand and H_2_ are polarized simultaneously, resulting in signals of opposite sign (Figure 1a). The hydrogen signal arises from the conversion of the parahydrogen, which is unobservable by NMR, to orthohydrogen as a byproduct of the SABRE process. Thus, the orthohydrogen contributes a signal to the measured relaxation data. The spin–spin relaxation rate of orthohydrogen at the high field was determined as *R*_2_ = 0.961 ± 0.022 s^–1^ (Table S8). The contribution of the orthohydrogen signal to the determined binding affinities at the low field was estimated under the assumption that its relaxation rate and relative signal amplitude are the same as those at the high field. The signal contribution of orthohydrogen is 4× smaller than the signal of the ligand in Figure 1a. To estimate the effect at the low field, the simulated integrals due to orthohydrogen were added to the measured data to estimate the net signal of the ligand (Figure S7). The resulting apparent change in relaxation is shown in Figure S8. Using these simulated signals, the *K*_D_ of the ligand (L) with trypsin results in 141.2 (138.6–142.7) μM, and the *K*_D,2_ of benzylamine with trypsin in 171 (140–154) μM (Table S7). The change in *K*_D_ for both the reporter ligand and benzylamine with trypsin is within the range of the values determined without considering the orthohydrogen signal. The minimal change in the determined *K*_D_ suggests that the effect of *o*-H_2_ on more than one of the measured relaxation rates resulted in a partial cancellation of the errors in the modeled concentrations.

## 4 Discussion

The binding interactions between the protein and ligand were interpreted from relaxation measurements by using SABRE hyperpolarization in a low-field magnet. The low-field NMR spectroscopy lacks chemical-shift resolution, but in contrast to conventional high-field NMR or even benchtop NMR, this measurement can be performed at a much lower cost. The polarization of the substrate produced by SABRE is several hundred times higher than the Boltzmann polarization in high-field NMR, leading to observable signals in the submillimolar concentration regime. The signal-to-noise ratio was 16.0 ± 3.5 for the dilution experiments at a ligand concentration of 1.67 ± 0.83 mM; thus, the hyperpolarized ligand would be detectable to 300 μM using the described instrument and hyperpolarization levels without any further optimizations (Table S9). This concentration is slightly higher than the 100 μM previously obtained using fluorine hyperpolarization.^25^ The protein concentration in the experiments was 33.33 ± 1.67 μM. This concentration could likewise be further reduced for binding experiments at lower ligand concentrations.

The measurement was achieved by detecting the ^1^H signal of an unlabeled ligand. Low-field NMR is not subject to interference from the nonhyperpolarized components of the sample; however, other hyperpolarized protons can contribute to the observed signal. Deuteration was used to remove signals from other potentially hyperpolarizable molecules that would overlap with the proton signal. The H_2_ signal that appears during the SABRE process still adds to the ligand signals, but it can be estimated and deducted to improve the calculation of the relaxation corresponding to the hyperpolarized ligand.

The SABRE was performed in methanol, where the catalyst with the 4,5-dimethyl-1,3-bis­(2,4,6-trimethylphenyl)­imidazole-2-ylidene ligand yielded optimal polarization. The 16.67% of *d*_4_-methanol present in the solution after dilution with the protein sample could be anticipated to have an effect on the protein stability and thus interfere with the protein–ligand interactions. The effect of the solvent on the trypsin was studied using an activity assay, monitoring the conversion of *N*-benzoyl arginine ethyl ester (BAEE). The reaction was performed under two different conditions—one in the aqueous buffer and the other with the same concentration of *d*_4_-methanol as in the experiment (Figure S9). A closely matching zero-order rate law, expected from the enzyme kinetics, was observed in both cases. The reaction slowed slightly earlier in the condition with methanol, but there was no significant difference observed during 10.2 s that corresponds to the time of the SABRE experiment. Therefore, the protein is expected to be in a nativelike conformation during the experiment. A previous study mixing *Tris* buffer at pH = 8.0 with several methanol concentrations reached the same conclusion.^19^

It is likely that different proteins would be affected to differing extents by the presence of methanol. Other optimizations of the solution conditions could be performed. For instance, some SABRE catalysts can be used directly in an aqueous solution. The lower solubility of the hydrogen gas and other efficiency constraints can further be overcome by a two-phase medium comprising reverse micelles.^32^ Similarly, the effect of the coligand for the polarization transfer catalyst on protein binding should be considered. Here, the coligand is not expected to bind strongly to trypsin, as it is a small molecule that does not contain an amidine moiety, and it is present at a lower concentration than the ligand.

The described implementation makes use of in situ hyperpolarization in the low-field NMR instrument, where the hyperpolarized ligand remains stationary, and the protein becomes injected. The mixing is caused by turbulence introduced by a sufficiently rapid injection. The observed *R*_2_ relaxation decays were fitted to single exponential curves expected for a homogeneously mixed solution without concentration gradients. The error range of the *R*_2_ fit parameters in almost all cases was smaller than the standard deviation of the values due to other experimental errors, indicating a close alignment of each curve with the exponential model. The NMR pulse sequence for the *R*_2_ measurement does not include pulsed field gradients and therefore is not directly affected by residual motions due to turbulent mixing in the NMR tube. Nevertheless, technical improvements of the experiment could include the use of a flow cell.^33^ A flow cell would be compatible with high-throughput analysis for screening of ligand binding, potentially allowing several hundred experiments per hour. From the experimental perspective, the use of a flow cell would also entail the external mixing of the protein and ligand solutions in a mixer device, which could be separately optimized.^19,25^ Other improvements for this application could include the development of heterogeneous catalyst systems,^34^ which ultimately may allow flow-through polarization of the ligand without consuming catalyst in each experiment.

In SABRE experiments, where signals are measured at high field, catalyst binding can overwhelmingly affect the relaxation rates. This exchange contribution is absent at low field. Thus, it is not necessary to deactivate the catalyst prior to the experiment. Additionally, in the low field, sample inhomogeneities such as foaming due to the protein injection do not affect the signals. The determination of protein and ligand binding using ^1^H detection of a reporter ligand broadens the application of low-field NMR to ligand screening in drug discovery.^7,25^ A key feature of this two-step paradigm involves the role of a SABRE hyperpolarized reporter ligand, where first the *K*_D_ of the reporter ligand is determined. Subsequently, the same reporter ligand is used for determining the affinity of any number of other, nonhyperpolarizable ligands of interest.

## 5 Conclusions

In conclusion, we have shown that low-field NMR with SABRE hyperpolarization of ^1^H nuclei can be used for the study of biological interactions between a ligand and protein. Spin–spin relaxation changes present a facile way to determine the binding affinity in an experiment, requiring only a single hyperpolarization step for each ligand. The detection of a protonated reporter ligand broadens the application of the platform for ligand screening based on SABRE hyperpolarization. The inclusion of a fluorine or other label is not necessary for observing the NMR signal, requiring only a ligand design with a functional group binding to the polarization transfer catalyst. The result shows the possibility of fragment screening for drug discovery or investigation of biochemical processes involving ligand binding by this method.

## Supplementary Material



## Notes

NMR spectroscopy; measurement of catalyst and ligand binding; measurement of protein–ligand binding; H_2_ signals; limit of detection; and trypsin activity assay (PDF)
