# Supplementary material for: Label-Free Measurement of Ligand Interactions Using SABRE Hyperpolarization at Low Magnetic Fields
Source: Anal Chem. 2026 Mar 10;98(11):8468–74. doi: 10.1021/acs.analchem.5c07983 (PMC13019426; doi:10.1021/acs.analchem.5c07983)
Supplement: Supplementary file 1 [file ac5c07983_si_001.pdf]

# Supporting Information

# Label-free measurement of Ligand Interactions using SABRE Hyperpolarization at Low Magnetic Fields

Ashes Roy, and Christian Hilty*

Chemistry Department, Texas A&M University, College Station, TX 77843, USA

*email: chilty@tamu.edu

**Table of Contents**

1. Abbreviations.......................................................S2
2. NMR Spectroscopy.......................................................S2
3. Measurement of Catalyst and Ligand Binding.......................................................S4
   a) Relaxation Measurements.......................................................S4
   b) Determination of [CL]/[L] Ratio for Various Catalyst Concentrations.......................................................S4
   c) Calculation of $R_{2,f}$ and $R_{2,CL}$.......................................................S5
4. Measurement of Protein-ligand Binding.......................................................S7
   a) Relaxation Measurements with Hyperpolarized Ligand.......................................................S7
   b) Relaxation Measurements with Hyperpolarized Ligand and Competing Ligands.......................................................S8
   c) Relaxation Equations for Protein Binding Experiments.......................................................S8
   d) Determination of $K_D$ of Hyperpolarized Reporter Ligand.......................................................S9
   e) Determination of $K_D$ of Benzylamine ($K_{D,3}$) as a Competing Ligand.......................................................S11
5. $H_2$ Signals.......................................................S12
   a) Relaxation Measurement of $H_2$.......................................................S12
   b) Change in Relaxation Measurement Due to Orthohydrogen Contribution.......................................................S12
6. Limit of Detection.......................................................S13
   Signal to Noise Ratio for Dilution Experiments.......................................................S13
7. Trypsin Activity Assay.......................................................S14
8. References.......................................................S14

# 1. Abbreviations

| | | |
|---|---|---|
| L | ligand | 3-amidinopyridine |
| CoL | co-ligand | dimethylsulfoxide-$d_6$ |
| C | (pre)-catalyst | Chloro(1,5-cyclooctadiene)[4,5-dimethyl-1,3-bis(2,4,6-trimethylphenyl)imidazol-2-ylidene]iridium(I) |
| P | protein | trypsin |
| S | competing ligand | benzamidine or benzylamine |

# 2. NMR Spectroscopy

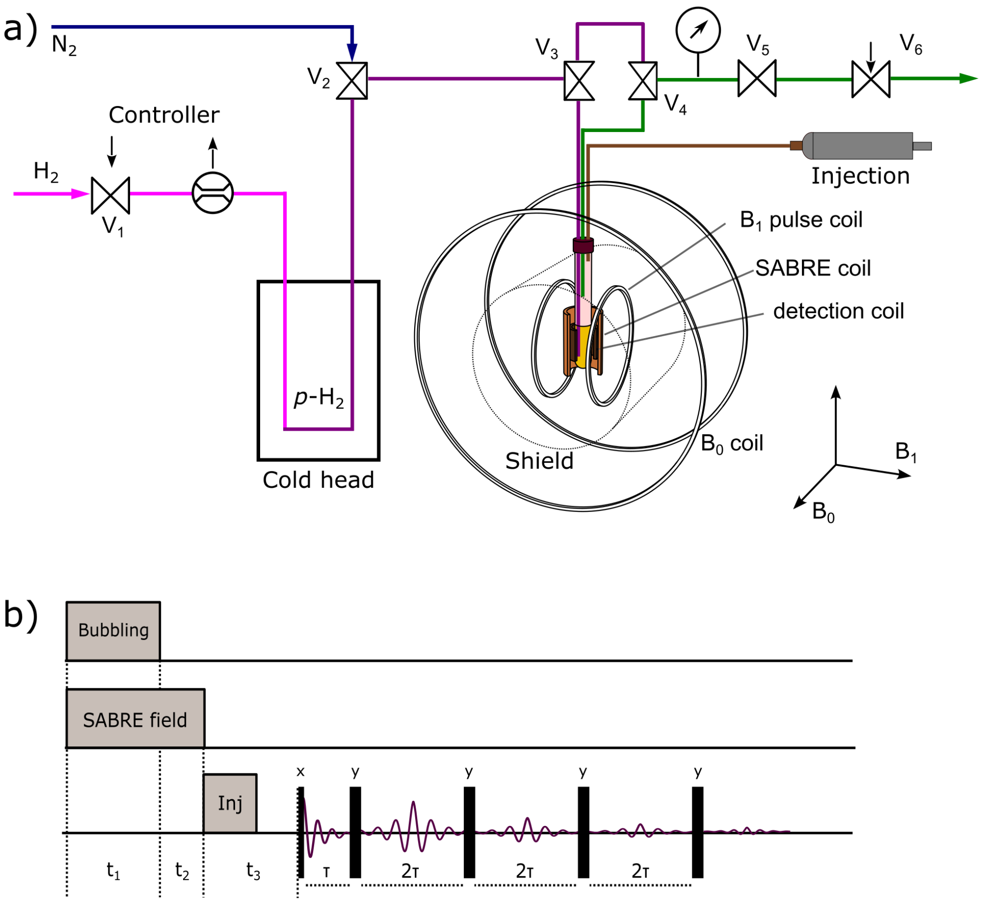


***Figure S1:*** *a) Schematic diagram of the low-field NMR setup (not to scale). $H_2$ is converted to parahydrogen at 29 K at the bottom of the cold-head and then is delivered to the sample. The valve $V_2$ allows flushing the sample with nitrogen (not used in the experiment). $V_3$ and $V_4$ allow bypassing the sample, and $V_6$ controls the flow of parahydrogen b) Pulse sequence for $R_2$ measurement. The time periods for bubbling of parahydrogen gas into the sample, enabling the constant magnetic field for polarization and injection of the protein or buffer sample component are indicated. Narrow and wide*

*black bars are π/2 and π pulses, respectively. The signal acquisition extends from the first pulse to the end of the pulse sequence. The appearance of signals is symbolized by the oscillating shapes. The phase of the 90º pulse was x and of the 180º pulses was y.*

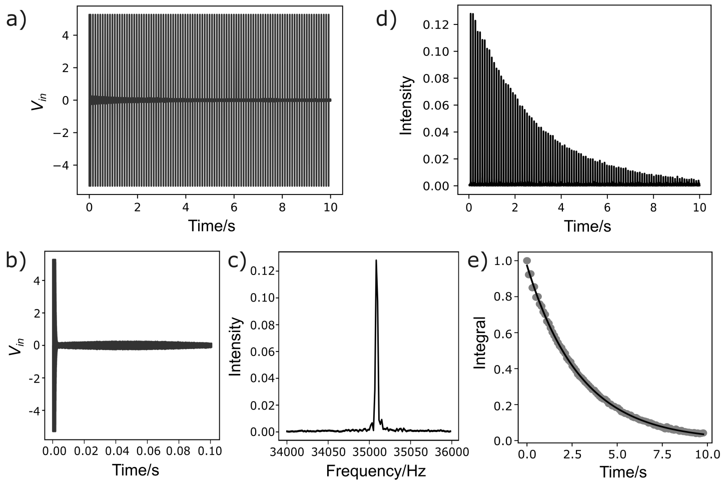


***Figure S2:*** *a) Time domain signal acquired with hyperpolarization of 3-amidinopyridine as ligand (L) in a sample containing Ir pre-catalyst (C), and dimethyl sulfoxide as co-ligand (CoL) using CPMG experiment. b) Time domain and c) frequency domain signal of the first echo from (a). d) Sequence of frequency domain signals from all echoes. e) Curve fitted to the integrals of (d). The sample contained 1.000 ± 0.050 mM of Chloro(1,5-cyclooctadiene)[4,5-dimethyl-1,3-bis(2,4,6-trimethylphenyl)imidazol-2-ylidene]iridium(I) as the pre-catalyst (C), 10.00 ± 0.50 mM of 3-amidino-pyridine hydrochloride as the ligand (L), and 10.00 ± 0.50 mM of $d_6$-DMSO as the co-ligand (CoL) in $d_4$-methanol.*[1,2]

# 3. Measurement of Catalyst and Ligand Binding

### a) Relaxation Measurements

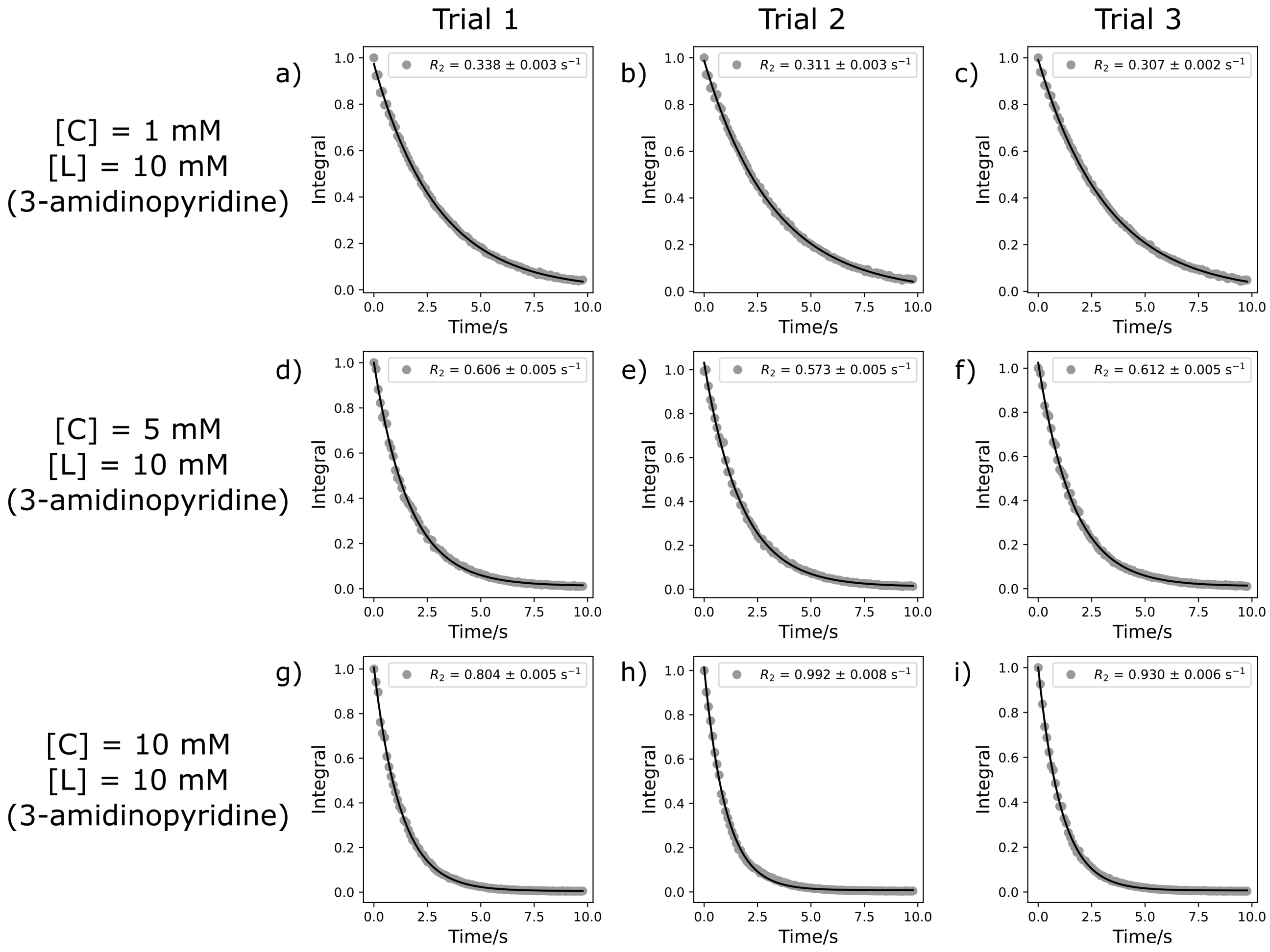


***Figure S3:*** *$R_2$ relaxation measurements of hyperpolarized ligand 3-amidinopyridine (L) with catalyst (C). a–c) 1.000 ± 0.050 mM C, 10.00 ± 0.50 mM L and 10.00 ± 0.50 mM CoL. d–f) 5.00 ± 0.25 mM C, 10.00 ± 0.50 mM L and 10.00 ± 0.50 mM CoL. g–i) 10.00 ± 0.50 mM C, 10.00 ± 0.50 mM L and 10.00 ± 0.50 mM CoL. The integrals from Fourier transformed spin echoes (•) are fitted to single exponential functions. The fitting errors listed with the individual curves are determined from the Jacobian matrix of the fitting procedure. The average values and the standard deviations from the three measurements for each sample condition are 0.319 ± 0.014 $s^{-1}$ to 0.597 ± 0.017 $s^{-1}$ to 0.909 ± 0.078 $s^{-1}$, respectively.*

### b) Determination of [CL]/[L] Ratio for Various Catalyst Concentrations

The concentration ratio of catalyst-bound ligand to the ligand in solution was calculated from the integral ratios of the corresponding signals in high-field NMR spectra (Figure S4). With an increasing catalyst concentration, the catalyst-ligand bound signal increases as shown in Table S1.

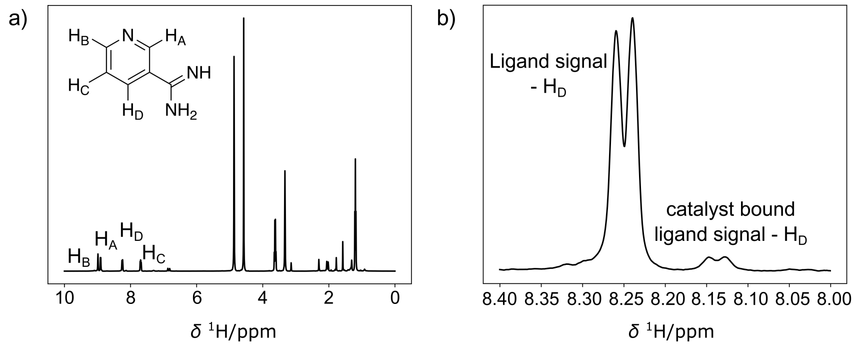


***Figure S4:*** *a) $^1$H NMR spectrum of a sample containing 1 mM C, 10 mM L and 10 mM CoL measured in a 400 MHz NMR spectrometer. b) Zoomed-in view of the ligand and catalyst bound ligand peaks for $H_D$. L is 3-amidinopyridine.*

***Table S1:*** *Concentration ratios of catalyst-bound ligand to free ligand at various catalyst concentrations. The samples contained 10 mM of L and 10 mM of CoL. The ratios were obtained from integration of NMR spectra measured at 400 MHz.*

| Catalyst Concentrations (mM) | Mean [CL]/[L] |
|---|---|
| 1 ± 0.05 mM | 0.0760 ± 0.0010 |
| 5 ± 0.25 mM | 0.1210 ± 0.0050 |
| 10 ± 0.5 mM | 0.16800 ± 0.00090 |

### c) Calculation of $R_{2,f}$ and $R_{2,CL}$

The relaxation rates of free and catalyst bound ligand, $R_{2,f}$, $R_{2,CL}$, and the equilibrium constant for the catalyst-ligand interaction, $K_{eq}$, were determined. A set of relaxation measurements of catalyst and ligand solutions with three different catalyst concentrations were performed. The dependence of the observed relaxation rates, $R_{2,obs}$, on concentration ratios are given by Eq. 2 in the main text. Additionally,

$$[L_{\text{tot}}] = [CL] + [L] \quad \text{(Eq. S1)}$$

$$[C_{\text{tot}}] = [CL] + [C] \quad \text{(Eq. S2)}$$

$$K_{\text{eq}} = \frac{[CL]}{[C][L]} \quad \text{(Eq. S3)}$$

Here, C is catalyst, L is ligand, CL is catalyst-ligand complex.

The best fitting solution to the equations with the input parameters in Table S2 was found numerically by minimizing the sum of residual squares using Python. Initial values for concentrations were chosen to be the total concentrations. For unknown parameters, initial values were zero. Bounds on

concentrations were set not to exceed initial concentrations. A Monte Carlo error analysis was performed by repeating the calculation 5000 times with randomized input values. The values followed Gaussian distributions with the standard deviations from the errors given in Table S2. The errors in the concentrations were assumed to be 5 percent. The standard deviations of the relaxation measurement and [$CL$]/[$L$] ratio were calculated from the three repetitions of the experiments. These standard deviations were larger than the propagated fitting errors from the three measurements, therefore the standard deviations were used in the calculations. The resulting calculated values are shown in Table S3.

***Table S2:*** *Input values for the minimization. The a, b, and c in the subscript indicate the 3 different sample conditions.*

| Parameters | Values |
|---|---|
| $[C_{\mathrm{tot}}^{(\mathrm{a})}]$ | 1.00 ± 0.05 mM |
| $[L_{\mathrm{tot}}^{(\mathrm{a})}]$ | 10.00 ± 0.50 mM |
| $[C_{\mathrm{tot}}^{(\mathrm{b})}]$ | 5.00 ± 0.25 mM |
| $[L_{\mathrm{tot}}^{(\mathrm{b})}]$ | 10.00 ± 0.50 mM |
| $[C_{\mathrm{tot}}^{(\mathrm{c})}]$ | 10.00 ± 0.50 mM |
| $[L_{\mathrm{tot}}^{(\mathrm{c})}]$ | 10.00 ± 0.50 mM |
| $[CL^{(\mathrm{a})}]/[L^{(\mathrm{a})}]$ | 0.0760 ± 0.0010 |
| $[CL^{(\mathrm{b})}]/[L^{(\mathrm{b})}]$ | 0.1210 ± 0.0050 |
| $[CL^{(\mathrm{c})}]/[L^{(\mathrm{c})}]$ | 0.16800 ± 0.00090 |
| $R_{2,\mathrm{obs}}^{(\mathrm{a})}$ | 0.319 ± 0.014 $s^{-1}$ |
| $R_{2,\mathrm{obs}}^{(\mathrm{b})}$ | 0.597 ± 0.017 $s^{-1}$ |
| $R_{2,\mathrm{obs}}^{(\mathrm{c})}$ | 0.909 ± 0.078 $s^{-1}$ |

***Table S3:*** *Output values from the minimization. The a, b, and c in the subscript indicate the 3 different sample conditions, where $R_{2,f}$ and $R_{2,CL}$ are relaxation corresponding to free ligand and catalyst-bound ligand.*

| Parameters | Values | Range |
|---|---|---|
| $[C^{(\mathrm{a})}]$ | 0.687 ± 0.088 mM | 0.518 – 0.803 mM |
| $[L^{(\mathrm{a})}]$ | 9.63 ± 0.38 mM | 9.05 – 10.00 mM |
| $[CL^{(\mathrm{a})}]$ | 0.33 ± 0.12 mM | 0.21 – 0.50 mM |

| | | |
|---|---|---|
| $[C^{(b)}]$ | 3.56 ± 0.42 mM | 2.83 – 4.17 mM |
| $[L^{(b)}]$ | 8.56 ± 0.53 mM | 7.66 – 9.44 mM |
| $[CL^{(b)}]$ | 1.44 ± 0.33 mM | 1.10 – 1.99 mM |
| $[C^{(c)}]$ | 7.41 ± 0.67 mM | 6.34 – 8.49 mM |
| $[L^{(c)}]$ | 7.40 ± 0.68 mM | 6.33 – 8.52 mM |
| $[CL^{(c)}]$ | 2.59 ± 0.56 mM | 1.89 – 3.44 mM |
| $R_{2,\mathrm{f}}$ | 0.231 ± 0.031 s$^{-1}$ | 0.17 – 0.28 s$^{-1}$ |
| $R_{2,\mathrm{CL}}$ | 2.92 ± 0.56 s$^{-1}$ | 1.93 – 3.85 s$^{-1}$ |
| $K_{\mathrm{eq}}$ | 0.0502 ± 0.0250 mM$^{-1}$ | 0.028 – 0.079 mM$^{-1}$ |

# 4. Measurement of Protein-ligand Binding

**a) Relaxation Measurements with Hyperpolarized Ligand**

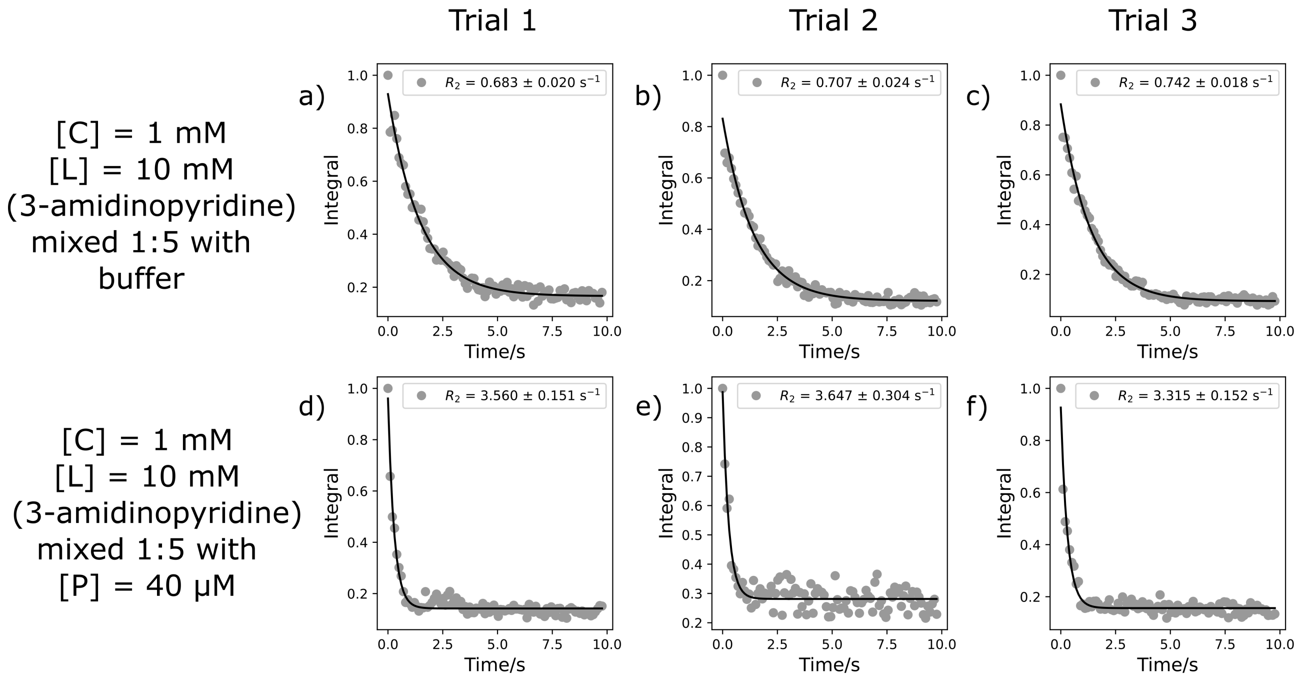


***Figure S5:*** *$R_2$ relaxation measurements of hyperpolarized ligand 3-amidinopyridine (L) with catalyst (C) and protein (P). a–c) 1.25 mL of phosphate buffer of pH 7.2 is mixed with the sample containing 0.25mL of 1.000 ± 0.050 mM C, 10.00 ± 0.50 mM L and 10.00 ± 0.50 mM CoL. d–f) 1.25 mL of 40 ± 2 μM trypsin is mixed with phosphate buffer of pH 7.2 to the sample containing 0.25 mL of 1.000 ± 0.050 mM C, 10.00 ± 0.50 mM L and 10.00 ± 0.50 mM CoL. The integrals from Fourier transformed spin echoes (•) are fitted to single exponential functions. The fitting errors listed with the individual curves are determined from the Jacobian matrix of the fitting procedure. The average values and the standard*

*deviations from the three measurements for each sample condition are 0.710 ± 0.024 $s^{-1}$ and 3.51 ± 0.14 $s^{-1}$, respectively.*

**b) Relaxation Measurements with Hyperpolarized Ligand and Competing Ligands**

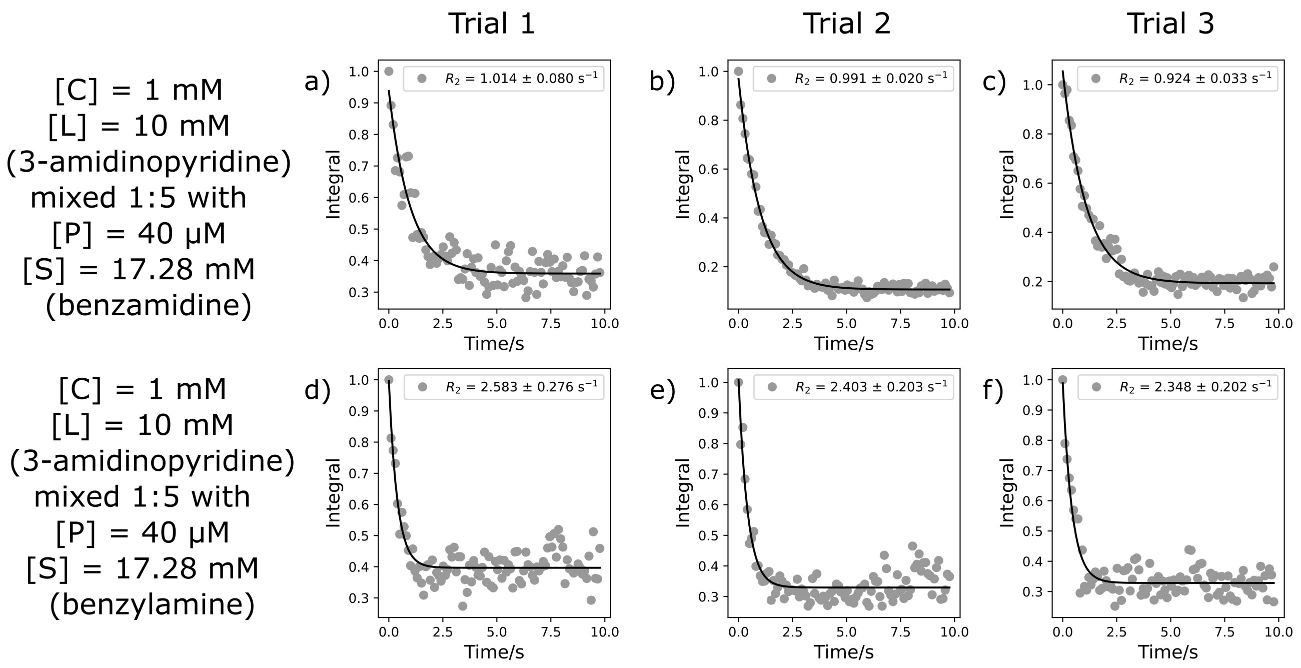


***Figure S6:*** *$R_2$ relaxation measurements of hyperpolarized ligand 3-amidinopyridine (L) with catalyst (C), protein (P) and competing ligand (S). a–c) 1.25 mL of 17.28 ± 0.864 mM benzamidine (competing ligand $S_a$) with 40 μM trypsin in phosphate buffer is mixed with the sample containing 0.25 mL of 1.000 ± 0.050 mM C, 10.00 ± 0.50 mM L and 10.00 ± 0.50 mM CoL; d–f) 1.25 mL of 17.28 mM benzylamine (competing ligand $S_b$) with 40 ± 2 μM trypsin in phosphate buffer is mixed with the sample containing 0.25 mL of 1.000 ± 0.050 mM C, 10.00 ± 0.50 mM L and 10.00 ± 0.50 mM CoL. The integrals from Fourier transformed spin echoes (•) are fitted to single exponential functions. The fitting errors listed with the individual curves are determined from the Jacobian matrix of the fitting procedure. The average values and the standard deviations from the three measurements for each sample condition are 0.975 ± 0.038 $s^{-1}$ and 2.444 ± 0.100 $s^{-1}$, respectively.*

**c) Relaxation Equations for Protein Binding Experiments**

***Protein-ligand binding***

The interpretation of experiments including protein and ligand binding requires the consideration of catalyst-ligand and protein-ligand equilibria. In addition to Eqs. S2 and S3, the calculation of the dissociation constant requires Eqs. S4-S6 along with Eq. 4. If the $K_D$ of the ligand is known, these equations are sufficient to calculate the relaxation rate of protein-bound ligand complex.

$$[L_{\mathrm{tot}}] = [CL] + [PL] + [L] \quad \text{(Eq. S4)}$$

$$[P_{\mathrm{tot}}] = [PL] + [P] \quad \text{(Eq. S5)}$$

$$K_{\mathrm{D}} = \frac{[P][L]}{[PL]} \quad \text{(Eq. S6)}$$

C is catalyst, L is ligand, CL is catalyst-ligand complex. P is protein, PL is protein-ligand complex

***Competitive protein-ligand binding***

The case of two competing ligands binding to a protein requires the consideration of catalyst-ligand, protein-ligand and protein-competitor ligand equilibria. In addition to Eqs. S2–S6, the calculation includes Eqs. S7 and S8 along with Eq. 4. With these equations, one of the dissociation constants can be calculated if the other one is known. Additionally, knowledge of the relaxation rate of the protein bound ligand is required.

$$K_{\mathrm{D},2} = \frac{[P][S]}{[PS]} \quad \text{(Eq. S7)}$$

$$[S_{\mathrm{tot}}] = [PS] + [S] \quad \text{(Eq. S8)}$$

S is competitor ligand, PS is protein-competitor ligand complex.

## d) Determination of $K_D$ of Hyperpolarized Reporter Ligand

The ligand 3-amidinopyridine will be used as a reporter ligand to determine the interaction of other ligands with the protein in competitive binding experiments. Since the $K_D$ of this ligand is not known, it was first determined using the measured data from the protein-ligand interaction (Figure S5d-f) combined with the competitive binding experiment with benzamidine (Figure S6a-c). These data sets were fitted using the above equations S2-S8 and Eq. 4 in the main text.

The calculation was performed in the same way as described in the preceding section with initial values calculated from the minimize function for accurate error measurement. The relaxation rates of free ligand and catalyst bound ligand were included from the results of the analysis in section 2d. The dissociation constant of benzamidine with trypsin, $K_{D,2}$ = 15.7 ± 1.9 μM from the literature was used.[3] The complete input values for the calculation are listed in Table S4, and the output values are in Table S5.

***Table S4:*** *Input values for the minimization, The 1 and 2 in the subscript indicate the different mixing conditions with protein, where 1 is P+L and 2 is P+L+S.*

| Parameters | Values |
|---|---|
| $[C_{\mathrm{tot}}^{(1)}]$ | 0.1667 ± 0.0083 mM |
| $[L_{\mathrm{tot}}^{(1)}]$ | 1.667 ± 0.083 mM |
| $[P_{\mathrm{tot}}^{(1)}]$ | 0.0333 ± 0.0016 mM |
| $[C_{\mathrm{tot}}^{(2)}]$ | 0.1667 ± 0.0083 mM |
| $[L_{\mathrm{tot}}^{(2)}]$ | 1.667 ± 0.083 mM |

| | |
|---|---|
| $[P_{tot}^{(2)}]$ | 0.0333 ± 0.0016 mM |
| $[S_{tot}]$ | 14.40 ± 0.72 mM |
| $K_{D,2}$ | 15.7 ± 1.9 μM |
| $R_{2,obs}^{(1)}$ | 3.51 ± 0.14 $s^{-1}$ |
| $R_{2,obs}^{(2)}$ | 0.976 ± 0.038 $s^{-1}$ |
| $R_{2,f}$ | 0.231 ± 0.031 $s^{-1}$ |
| $R_{2,CL}$ | 2.92 ± 0.561 $s^{-1}$ |
| $K_{eq}$ | 0.0502 ± 0.0250 $mM^{-1}$ |

***Table S5:*** *Output values from the minimization. The 1 and 2 in the subscript indicate the different mixing conditions with protein, where 1 is P+L and 2 is P+L+S, and $R_{2,PL}$ is the relaxation of protein-bound ligand complex.*

| Parameters | Values | Range |
|---|---|---|
| $[L^{(1)}]$ | 1.609 ± 0.058 mM | 1.521 – 1.667 mM |
| $[C^{(1)}]$ | 0.148 ± 0.015 mM | 0.126 – 0.167 mM |
| $[P^{(1)}]$ | 0.00256 ± 0.00044 mM | 0.0018 – 0.0031 mM |
| $[CL^{(1)}]$ | 0.020 ± 0.016 mM | 0.000 – 0.046 mM |
| $[PL^{(1)}]$ | 0.0292 ± 0.0051 mM | 0.022 – 0.033 mM |
| $[C^{(2)}]$ | 0.145 ± 0.019 mM | 0.116 – 0.167 mM |
| $[L^{(2)}]$ | 1.626 ± 0.052 mM | 1.541 – 1.667 mM |
| $[P^{(2)}]$ | 0.0000366 ± 0.0000047 mM | 0.000028 – 0.000043 mM |
| $[S^{(2)}]$ | 14.09 ± 0.43 mM | 13.42 – 14.40 mM |
| $[CL^{(2)}]$ | 0.024 ± 0.021 mM | 0.000 – 0.059 mM |
| $[PL^{(2)}]$ | 0.00045 ± 0.00086 mM | 0.00000 – 0.00067 mM |
| $[PS^{(2)}]$ | 0.0328 ± 0.0015 mM | 0.031 – 0.033 mM |
| $R_{2,PL}$ | 35.2 ± 1.1 $s^{-1}$ | 33.8 – 35.3 $s^{-1}$ |
| $K_D$ | 141.1 ± 6.7 μM | 138.9 – 143.1 μM |

## e) Determination of $K_D$ of Benzylamine ($K_{D,3}$) as a Competing Ligand

The determination of a ligand binding affinity using 3-amidinopyridine as a reporter ligand is demonstrated using the data in Figure S6d-f. The competing ligand is benzylamine. With the above equations S2-S8 and Eq. 4, the dissociation constant for benzylamine, $K_{D,3}$, is calculated. The input values are in Table S6 and output value in Table S7. In these measurements, the relaxation rates of free ligand and catalyst bound ligand complexes have been considered from the results of the analysis in section 3c, and the relaxation rate of protein bound reporter ligand is from section 4c. The calculation was performed in the same way as described in the preceding section. As the only exception, the propagated fitting error for $R_{2,obs}$ was larger than the standard deviation of the values, and was used for the calculation. Bounds on concentrations were set not to exceed initial concentrations. A large boundary range from 0 to 700 μM for the dissociation constant of benzylamine was applied.[4]

The resulting dissociation constant for benzylamine of 167 ± 92 μM compares to the literature of 200 ± 80 μM.[3,5]

***Table S6:*** *Input values for the minimization. (*) is error propagated from fitted values.*

| Parameters | Values |
|---|---|
| $[C_{tot}]$ | 0.1667 ± 0.0083 mM |
| $[L_{tot}]$ | 1.667 ± 0.083 mM |
| $[P_{tot}]$ | 0.0333 ± 0.0016 mM |
| $[S_{tot}]$ | 14.40 ± 0.72 mM |
| $K_D$ | 141.1 ± 6.7 μM |
| $R_{2,obs}$ | 2.44 ± 0.13 $s^{-1}$ (*) |
| $R_{2,f}$ | 0.231 ± 0.031 $s^{-1}$ |
| $R_{2,CL}$ | 2.92 ± 0.561 $s^{-1}$ |
| $R_{2,PL}$ | 35.2 ± 1.1 $s^{-1}$ |
| $K_{eq}$ | 0.0502 ± 0.0250 $mM^{-1}$ |

***Table S7:*** *Output values from the minimization.*

| Parameters | Values | Range |
|---|---|---|
| $[C]$ | 0.147 ± 0.020 mM | 0.118 – 0.167 mM |
| $[L]$ | 1.627 ± 0.053 mM | 1.541 – 1.667 mM |

| | | |
|---|---|---|
| $[P]$ | 0.00038 ± 0.00022 mM | 0.00028 – 0.00037 mM |
| $[S]$ | 14.10 ± 0.43 mM | 13.43 – 14.40 mM |
| $[CL]$ | 0.022 ± 0.021 mM | 0.000 – 0.054 mM |
| $[PL]$ | 0.0044 ± 0.0027 mM | 0.0029 – 0.0045 mM |
| $[PS]$ | 0.0316 ± 0.0022 mM | 0.028 – 0.033 mM |
| $K_{D,2}$ | 170 ± 101 μM | 140 – 154 μM |

# 5. $H_2$ Signals

### a) Relaxation Measurement of $H_2$

The signal contribution of orthohydrogen was estimated from high-field NMR measurements. Relaxation rates measured are listed in the Table S8.

***Table S8:*** *Relaxation measurements at 400 MHz $^1$H NMR frequency for orthohydrogen in a sample containing 1.00 ± 0.05 mM C, 10.0 ± 0.5 mM L and 10.0 ± 0.5 mM CoL in deuterated methanol.*

| Measurement | $R_2$ ($H_2$) | Average |
|---|---|---|
| 1 | 0.982 $s^{-1}$ | |
| 2 | 0.969 $s^{-1}$ | 0.961 ± 0.022 $s^{-1}$ |
| 3 | 0.931 $s^{-1}$ | |

### b) Change in Relaxation Measurement Due to Orthohydrogen Contribution

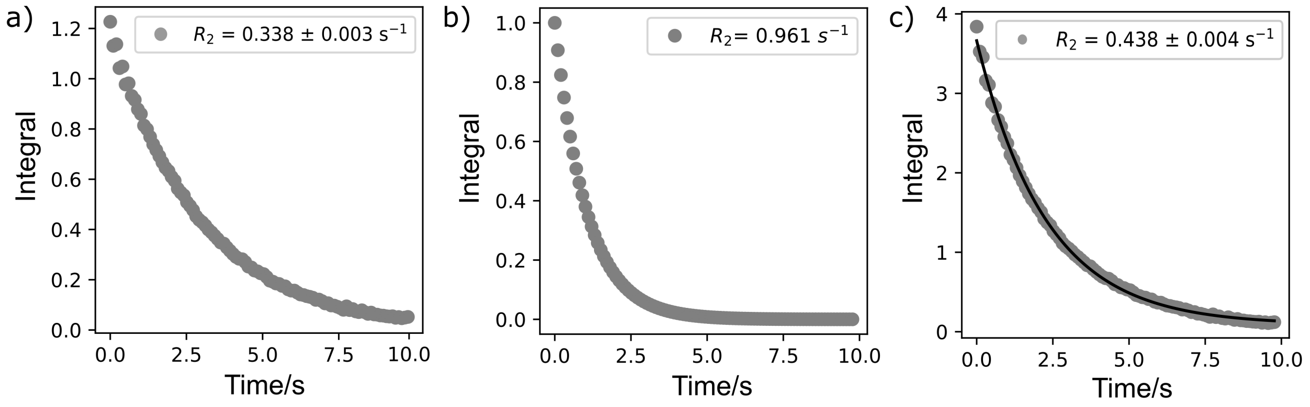


***Figure S7:*** *a) Measured data points of a sample containing 1.00 ± 0.05 mM C, 10.0 ± 0.5 mM L, and 10.0 ± 0.5 mM CoL. The fitting error listed with the curve is determined from the Jacobian matrix of*

*the fitting procedure. b) Simulated data points from the measured relaxation of orthohydrogen in the same time frame as (a). c) Generated data points corresponding to ligand signal only from the addition of (a) and (b). L is 3-amidinopyridine.*

The orthohydrogen contributes approximately 26 percent to the total $^1$H signal in the high-field NMR spectrum in Figure 1a. The error in the calculation of the relaxation rates at the low field is estimated by assuming the same contribution. Data points corresponding to orthohydrogen in the relaxation curve were simulated (Figure S7b). The simulated data is added to the measured low-field NMR data points (Figure S7a) to formally remove the orthohydrogen contribution, which has an opposite sign compared to the other signals. The relaxation calculated from the resulting points is shown in Figure S7c.

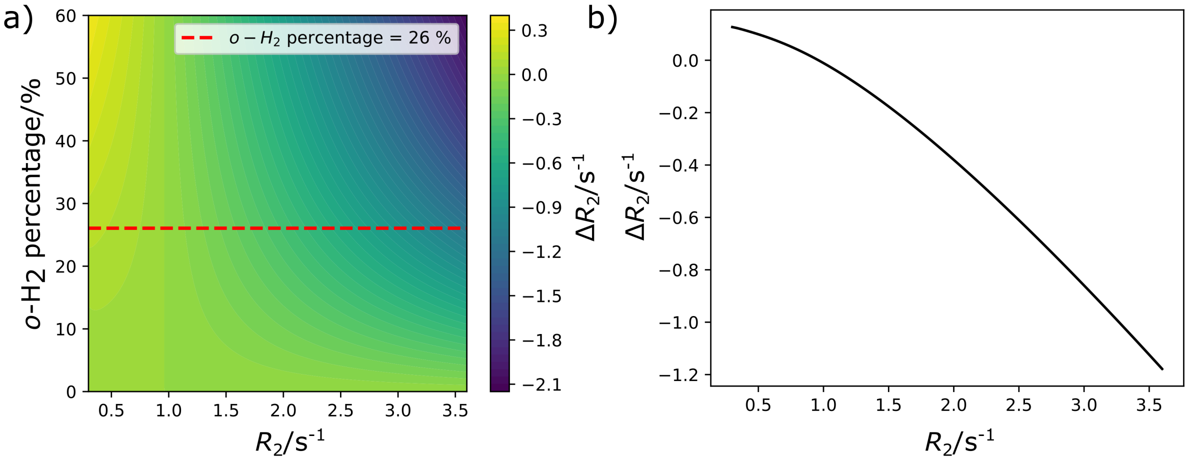


***Figure S8:*** *a) Contour plot showing the change in observed relaxation rate, $\Delta R_2$, for a range of orthohydrogen signal contribution percentage on the vertical axis, and the $R_2$ of the ligand on the horizontal axis. b) Plot of $\Delta R_2$ with respect to $R_2$ of the ligand for 26% orthohydrogen, which is similar to that of the experiment.*

Figure S8 shows the relaxation change over a range of measured relaxation rates. From this figure it is evident that change in ligand relaxation due to orthohydrogen contribution, $\Delta R_2$, is high in magnitude for high values of $R_2$. Further, the $\Delta R_2$ is positive when $R_2$ is less than the relaxation rate of orthohydrogen and negative in the opposite case. The reason is that the addition of orthohydrogen contribution makes the relaxation values closer to the value of relaxation of orthohydrogen.

# 6. Limit of Detection

### Signal to Noise Ratio for Dilution Experiments

***Table S9:*** *Ligand (L) Signal to noise ratio of the first echo calculated for the 3 sets of measurements when 0.25 mL of 1.00 ± 0.05 mM C, 10.0 ± 0.5 mM L and 10.0 ± 0.5 mM CoL was diluted with 1.25 mL of phosphate buffer.*

# 7. Trypsin Activity Assay

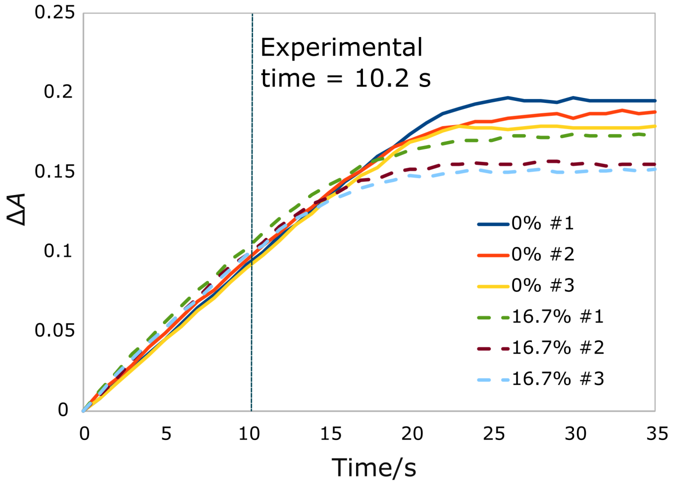


***Figure S9:*** *Change in absorbance (ΔA) at 253 nm vs. time for a reaction of 0.895 μM trypsin and 0.5 mM N-benzoyl arginine ethyl ester (BAEE) in 0% and 16.67% $d_4$-methanol.*

A kinetic assay of the reaction between trypsin protein and N-benzoyl arginine ethyl ester (BAEE) was performed to determine the stability of trypsin under the experimental conditions.[6] The change in absorbance in Figure S9 shows the formation of the product BA (N-benzoyl arginine). The kinetics of the reaction for the first 15 seconds is closely matching with and without $d_4$-methanol for the 10.2 s experimental duration showing the stability of protein during the experiment. A similar observation was made in ref. [7] for different experimental conditions.

# 8. References


(1) Pham, P.; Hilty, C. Biomolecular Interactions Studied by Low-Field NMR Using SABRE Hyperpolarization. *Chem. Sci.* **2023**, *14* (37), 10258–10263. https://doi.org/10.1039/D3SC02365F.

(2) Pham, P.; Hilty, C. $R_2$ Relaxometry of SABRE-Hyperpolarized Substrates at a Low Magnetic Field. *Anal. Chem.* **2023**, *95* (46), 16911–16917. https://doi.org/10.1021/acs.analchem.3c02709.

(3) Kim, Y.; Hilty, C. Affinity Screening Using Competitive Binding with Fluorine-19 Hyperpolarized Ligands. *Angew. Chem. Int. Ed.* **2015**, *54* (16), 4941–4944. https://doi.org/10.1002/anie.201411424.

(4) Ionides, E. L. Truncated Importance Sampling. *J. Comput. Graph. Stat.* **2008**, *17* (2), 295–311.

(5) Mandal, R.; Pham, P.; Hilty, C. Screening of Protein–Ligand Binding Using a SABRE Hyperpolarized Reporter. *Anal. Chem.* **2022**, *94* (32), 11375–11381. https://doi.org/10.1021/acs.analchem.2c02250.

(6) Crowell, A. M. J.; Stewart, E. J.; Take, Z. S.; Doucette, A. A. Critical Assessment of the Spectroscopic Activity Assay for Monitoring Trypsin Activity in Organic-Aqueous Solvent. *Anal. Biochem.* **2013**, *435* (2), 131–136. https://doi.org/10.1016/j.ab.2012.12.019.

(7) Mandal, R.; Pham, P.; Hilty, C. Characterization of Protein–Ligand Interactions by SABRE. *Chem. Sci.* **2021**, *12* (39), 12950–12958. https://doi.org/10.1039/D1SC03404A.
